# The constancy of global regulation across a species: the concentrations of ppGpp and RpoS are strain-specific in *Escherichia coli*

**DOI:** 10.1186/1471-2180-11-62

**Published:** 2011-03-25

**Authors:** Thomas Ferenci, Heloisa Filus Galbiati, Thu Betteridge, Katherine Phan, Beny Spira

**Affiliations:** 1 Departamento de Microbiologia, Instituto de Ciências Biomédicas, Universidade de São Paulo, São Paulo-SP, Brazil; 2 School of Molecular and Microbial Biosciences, The University of Sydney, NSW 2006 Australia

## Abstract

**Background:**

Sigma factors and the alarmone ppGpp control the allocation of RNA polymerase to promoters under stressful conditions. Both ppGpp and the sigma factor σ^S ^(RpoS) are potentially subject to variability across the species *Escherichia coli*. To find out the extent of strain variation we measured the level of RpoS and ppGpp using 31 *E. coli *strains from the ECOR collection and one reference K-12 strain.

**Results:**

Nine ECORs had highly deleterious mutations in *rpoS*, 12 had RpoS protein up to 7-fold above that of the reference strain MG1655 and the remainder had comparable or lower levels. Strain variation was also evident in ppGpp accumulation under carbon starvation and *spoT *mutations were present in several low-ppGpp strains. Three relationships between RpoS and ppGpp levels were found: isolates with zero RpoS but various ppGpp levels, strains where RpoS levels were proportional to ppGpp and a third unexpected class in which RpoS was present but not proportional to ppGpp concentration. High-RpoS and high-ppGpp strains accumulated *rpoS *mutations under nutrient limitation, providing a source of polymorphisms.

**Conclusions:**

The ppGpp and σ^S ^variance means that the expression of genes involved in translation, stress and other traits affected by ppGpp and/or RpoS are likely to be strain-specific and suggest that influential components of regulatory networks are frequently reset by microevolution. Different strains of *E. coli *have different relationships between ppGpp and RpoS levels and only some exhibit a proportionality between increasing ppGpp and RpoS levels as demonstrated for *E. coli *K-12.

## Background

Sigma factors direct RNA polymerase to various sets of promoters, and are at the centre of complex networks regulating gene expression in bacteria such as *Escherichia coli *[1,2]. Sigma factors are highly conserved in comparison to more specific regulators [1], but does genetic conservation imply functional conservation at the core of cell regulation? This is an important question in light of current systems biology efforts to construct models of regulatory behaviour [3-5]. There are instances where regulation differs between closely related bacteria [6-8] so how conserved is regulation, especially global regulation, within a species? We approach this question by measuring the concentration of two cellular components with global regulatory roles in multiple members of the same species. We focus on two factors with complementary functions in switching between vegetative growth and stress-related gene expression. The RpoS sigma factor (σ^S^), responds to stress and shifts transcription away from vegetative growth and towards stress resistance [9-12]. Higher levels of RpoS in stressed or stationary-phase cells alter expression of several hundred genes [13,14]. The alarmone ppGpp [15] also accumulates in bacteria undergoing stress, such as amino acid, carbon or phosphate limitation [16-19]. Accumulation of ppGpp triggers the stringent response and a radical decrease in ribosome and protein synthesis, even leading to growth arrest [20,21]. ppGpp and σ^S ^co-operate both mechanistically and strategically under stress and expression of σ^S^-controlled genes is partly dependent on ppGpp [22,23]. The level of ppGpp also controls the amount of σ^S ^in the cell, as ppGpp increases by several-fold the cellular concentration of σ^S ^during nutritional stress or in the stationary phase. The absence of ppGpp impairs or severely delays the accumulation of σ^S ^[9] and ppGpp positively affects the efficiency of *rpoS *translation under stress conditions as well as *rpoS *basal expression under conditions of optimal growth [24,25]. The response to phosphate starvation additionally involves stabilisation of RpoS protein sensed through SpoT [19]. At several levels then, ppGpp is intertwined with *rpoS *regulation and here we investigate the conservation of the level of these regulators across the species E. coli.

This study was prompted by several indications that RpoS and ppGpp were subject to strain variation. The *rpoS *gene is polymorphic in isolates of *E. coli *[26]. Recently, variations in ppGpp levels were also observed between laboratory strains of *E. coli *due to *spoT *mutations [21]. However, the assumption that *rpoS *is subject to extensive variation has been challenged [27]. These authors claimed that the endogenous RpoS levels are actually fairly conserved in *E. coli*. They also noted that the trade-off hypothesis was originally based on only two high-RpoS strains in [28].

Here, we study the hypothesis that stress-related gene expression is variable across the species *E. coli *because it involves a trade-off in the expression of genes related to stress resistance and vegetative growth [11]. The equilibrium between metabolic capacity essential during vegetative growth and stress resistance, the so-called SPANC (Self Preservation and Nutritional Competence) balance [11], is subject to selection in laboratory culture [28]. High levels of σ^S ^impair the growth of *E. coli *on poor carbon sources or under nutrient limitation [28]. Stress resistance is not constant amongst all *E. coli *strains [28-30] also indicating possible variation in gene expression relating to RpoS and/or ppGpp. We demonstrate here that strain variation in ppGpp is one of several factors that contribute to the difference in the level of σ^S ^across the species *E. coli *and discuss the polymorphisms at the core of bacterial regulation.

## Results

The goal of this study is three-fold: to provide evidence that *rpoS *polymorphism and variation in σ^S ^levels are widespread in the species *E. coli*; to show that the genes that control ppGpp synthesis and degradation are also subject to variation and finally to demonstrate that the different levels of RpoS are at least partially dependent on variability of endogenous ppGpp.

### Strain variation in RpoS levels in the species *E. coli*

**Figure 1:**
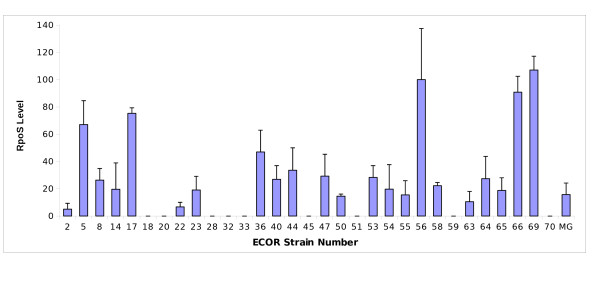
**Quantitation of RpoS**. Overnight bacterial cultures grown in LB were harvested, lysed and their total protein content resolved by SDS-PAGE. Proteins were immunoblotted with anti-RpoS monoclonal antibodies. The bands were scanned and quantified. Densitometric measurements were normalised against ECOR 56 to which was assigned 100 units. Relative values represent the mean ± S.E. of at least three independent experiments.

To test the extent of variation in RpoS levels, we analysed 31 strains from the ECOR collection of *E. coli *isolates from various locations and environments [31]. The 72 ECOR strains are divided into five phylogenetic groups (A, B1, B2, D and E). Nine of the strains tested here belonged to group A, 7 to group B1, 10 to group B2 and 5 to group D. The K-12 strain MG1655 was used as a control reference. As shown in Figure 1, the cellular content of RpoS was highly variable in standardised overnight cultures. Nine isolates had no detectable RpoS, another five had RpoS level 3 to 7-fold above that of the laboratory K-12 strain MG1655. The remainder of strains had levels within a 2-fold range around MG1655. The absence of RpoS from the nine strains was confirmed by screening for σ^S^-related phenotypes (glycogen accumulation [32] and catalase activity [33]; results not shown).

### *rpoS *sequences in ECOR strains

Variation in the *rpoS *locus was already indicated by the observation that PCR amplification of the *rpoS *region resulted in fragments of three different sizes, as shown in Table 1. These differences were consistent with the genomic variation in the *rpoS-mutS *region in the species *E. coli *[34]. The size of fragments and sequence matches correspond to previously described *rpoS *regions, with the 1.3 Kb fragment like that in *E. coli *K-12, and the 4.2 Kb and 3.4 Kb products similar to those found in [35] and [36] respectively. Sequencing of the *rpoS *gene in 22 ECOR strains (Table 1) representing high, low and null RpoS phenotypes indicated highly deleterious mutations (nonsense or frameshift) resulting in stop codons in *rpoS *in all ECORs with no detectable RpoS. That nearly a third of strains carried mutations in *rpoS *is striking, but not inconsistent with previous data with other *E. coli *strains. Bhagwat et al. [37] found that an introduced plasmid with wild-type *rpoS *was able to restore resistance in 20 acid-sensitive isolates amongst 82 pathogenic *E. coli *isolates tested. Similar results were obtained by [38]. Hence *rpoS*-defective strains consistently constitute 20-30% of natural isolates.

**Table 1:** Sequence analysis of *rpoS *in twenty-two ECOR strains

Strain	^**a**^***rpoS *PCR fragment size**	^**b**^**Change in nucleotide sequence**	^**b**^**Change in amino acid sequence**
ECOR02	1.3 Kb	C97G	Q33E

ECOR05	1.3 Kb	C97G,C942T	Q33E

ECOR08	1.3 Kb	C97G,C942T	Q33E

ECOR17	1.3 Kb	C97G, G377T, C942T	Q33E, G126V

ECOR18	1.3 Kb	C97G, ΩT392, C942T	Q33E, E132R, K133E, F134V, D135 amber *

ECOR20	1.3 Kb	T32G, C97G, C942T	L11 amber, Q33E *

ECOR22	1.3 Kb	C97G, C777T, C942T	Q33E

ECOR28	4.2 Kb	ΩA269	Frameshift after aa R85 *

ECOR32	4.2 Kb	C97G,G598T	Q33E, E200amber *

ECOR33	4.2 Kb	C97G, ΩA after nt494, ΩT after nt915	Q33E, frameshift after I165 *

ECOR45	4.2 Kb	ΩA518	Frameshift after aa 174 *

ECOR50	4.2 Kb	C264T, T270C, T357G, T462C, T549C, G564A, T573C, G819A	wild type

ECOR51	3.4 Kb	ΩT76, C97G,T163C, C264T, T357G, T462C, T573C, C732T, G819A, C987T	D26 amber *

ECOR54	3.4 Kb	ΩA after nt83, C97G, T163C, C264T, T357G, T462C, T573C, C732T, G819A, C987T	Q33E, frameshift after K28**

ECOR55	3.4 Kb	C97G, T163C, C264T, T357G, T462C, T573C, C732T, G819A, C987T	Q33E

ECOR56	3.4 Kb	C97G, T163C, T357G, G377A, T462C, T573C, C732T, G819A, C987T	Q33E, G126E

ECOR58	4.2 Kb	C97G, C672T	Q33E

ECOR59	3.4 Kb	C97G, G124T, T163C, T339C, T357G, C405T, T462C, T573C, C732T	Q33E, E42 amber and frameshift after aa S186 *

ECOR63	3.4 Kb	C97G, T163C, T357G, C405T, T462C, T573C, C732T, G990A	Q33E

ECOR66	3.4 Kb	C97G, T163C, T357G, C421T, T462C, T573C, C732T	Q33E, R141C

ECOR69	4.2 Kb	C97G	Q33E

ECOR70	1.3 Kb	Δnt94-nt121 (28nts)	Δaa32-41 (10aas) *

^a ^The PCR product covering the *rpoS *gene was of differing size, consistent with variation in the *rpoS-mutS *region in the species *E. coli *[34]. The 1.3 Kb fragment corresponds to *E. coli *K-12, and the 4.2 Kb and 3.4 Kb products are equivalent to regions found by [35,36].

^b ^The comparison is to the *E. coli *K-12 *rpoS *sequence

* Not detectable RpoS in immunoblots (see Figure 1)

** Truncated RpoS, as described [63]

The strains with high levels of RpoS were also sequenced for *rpoS*, but were mainly similar to the K-12 sequence. As shown in Table 1, several contained the commonly observed Q33E difference found amongst many K-12 strains but which has similar functional activity [39]. There is a G126 substitution to E or V in two of the five strains with high RpoS, but the significance of this is not clear. Two isolates with very low RpoS levels (ECOR2, ECOR22) had the same amino acid sequence as the strain with highest protein (ECOR69) so the structural gene is not the essential cause of RpoS variation. Given the many regulatory inputs affect RpoS protein levels [40], this is not altogether surprising; for example an *rssB *mutation can elevate RpoS level in some lab lineages [41].

### RpoS loss in ECOR strains

**Figure 2:**
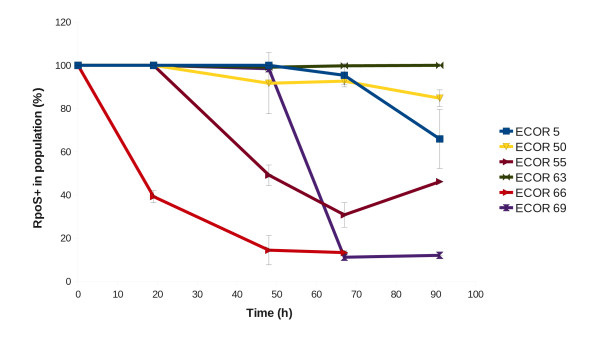
**The rate of acquisition of *rpoS *mutations in nutrient-limited chemostats**. ECOR strains were inoculated into glucose-limited chemostats and culture samples were withdrawn every 24 h for 4 days as previously described [32]. The aerobic chemostat populations were supplied with 0.02% glucose at a pH of 7, a temperature of 37°C and operating at a dilution rate of 0.1 h^-1^. The lines represent the proportion of wild-type bacteria, and the error bars on points show the standard deviations between two replicate chemostats with each strain. RpoS levels of tested strains (data from Figure 1): ECOR5 (67.1); ECOR50 (14.5); ECOR55 (15.5); ECOR63 (10.5); ECOR66 (90.8); ECOR69 (107.0).

The high level of σ^S ^in K-12 strains such as MC4100TF is associated with a measurably greater incidence of *rpoS *mutations in nutrient-limited populations than found with low- σ^S ^strains like MG1655 [28]. To see if the elevated RpoS in ECOR strains increased the selection pressure for *rpoS *mutations under nutrient limitation, the spread of *rpoS *mutations was followed in chemostat cultures limited by glucose, with all cultures growing at the same rate (μ = 0.1 h^-1^). The rate of enrichment of *rpoS *mutations in Figure 2 showed that strains with higher levels (ECOR66, 69) accumulated significant numbers of *rpoS *mutations within three days of continuous culture. With some intermediate-level strains, *rpoS *mutations still proliferated in the culture, but more slowly. There was no absolute relationship between RpoS level and rate of *rpoS *sweeps because one strain (ECOR5) had fairly high σ^S ^but the culture accumulated mutations slowly, while another (ECOR55) had low- σ^S ^levels but the culture rapidly accumulated *rpoS *mutations. As in earlier data, MG1655 did not accumulate mutations in *rpoS *under these conditions [28]. Hence it is evident that mutational changes can generally reassort RpoS levels in certain environments but differences between the strains besides RpoS levels need to be invoked to explain the extent of *rpoS *changes under glucose limitation. A possible difference is in the level of other global regulators affecting σ^S ^synthesis or degradation; below we investigate the variation in ppGpp as a possible contributor to RpoS variation.

### Strain variation in ppGpp levels in the species *E. coli*

**Figure 3:**
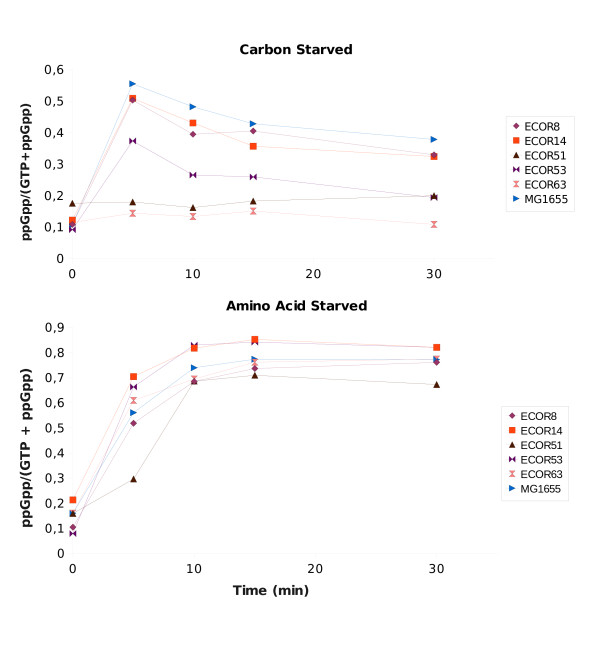
**Kinetics of ppGpp accumulation in ECOR strains starved for carbon or amino acid**. ^32^P-labelled cultures of exponentially-growing cells were treated with 2% α-MG (to induce carbon starvation) or 1 mg/ml SH (to induce amino acid starvation). Samples were withdrawn at time intervals and assayed for ppGpp. Values represent the level of ppGpp relative to GTP + ppGpp.

Recent experiments with laboratory strains [21] suggested that ppGpp levels were under SPANC selection and likely to be subjected to frequent microevolution under stress or under nutrient limitation. Initial experiments on some ECOR strains showed the kinetics of accumulation of ppGpp upon amino acid starvation (elicited by serine hydroxamate [42]) and carbon starvation (elicited by the addition of the inhibitor methyl-α-glucoside (α-MG [43]) were distinct. Amino acid starvation mainly operates through RelA and the level of ppGpp accumulation was quite similar in all strains (Figure 3b). In contrast in Figure 3a, it is evident that ppGpp response under carbon starvation was much more heterogeneous, consistent with variations in SpoT or its regulation by carbon starvation.

**Figure 4:**
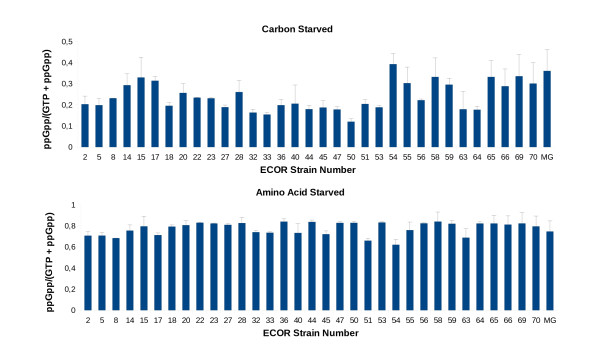
**ppGpp levels of ECOR strains starved for carbon or amino acid**. Cells were treated as in the legend of Figure 3, except that samples were withdrawn 30 minutes following the addition of α-MG or SH. ECORs 50, 51, 53 and 63 carry a T13N substitution in *spoT*. Bars represent the mean ± SD of three independent measurements.

Based on the kinetics in Figure 3, the level of ppGpp appeared to stabilise at around 30 min (in agreement with [44]) and a 30 min point was used to survey other ECOR strains. The levels of ppGpp measured under carbon starvation and amino acid starvation respectively are shown in Figure 4a and 4b. Overall, the stringent response with amino acid starvation was present and relatively constant in all strains (collective mean = 0.78, SD = 0.06, SD/mean = 0.08). On the other hand, the ppGpp levels triggered by α-MG addition varied over a much greater range (collective mean = 0.24, SD = 0.07, SD/mean = 0.29), consistent with the more heterogeneous kinetics in Figure 3.

DNA sequencing of the *spoT *gene from four high- and four low-ppGpp strains in Figure 4 revealed a mutation common in several low-ppGpp strains. A T13N substitution not present in lab strains or high-ppGpp strains was found in ECOR50, 51, 53 and 63. Although there is no direct evidence implicating these substitutions in altered ppGpp levels, these polymorphisms and those found in laboratory strains [21] are possibly consistent with *spoT *being subject to microevolutionary pressures.

### The relationship between ppGpp and RpoS levels in the species *E. coli*

**Figure 5:**
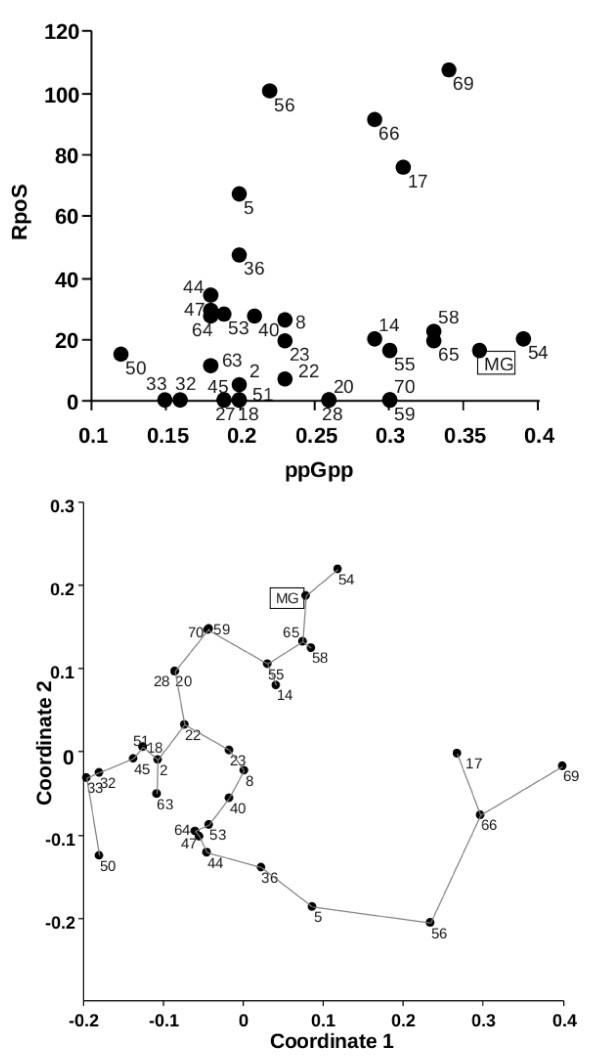
**The relationship between ppGpp and RpoS concentration in bacteria**. (a) A plot of the RpoS concentration against ppGpp concentration for the numbered ECOR isolates. (b) Multivariate analysis was performed using non-metric multidimensional scaling and Gower similarity measures using the software *Past *[62]. The lines between points show the minimum spanning tree drawn by the program.

As shown in Figure 5a, a plot of the measured ppGpp and RpoS levels in all the strains does not give a simple relationship in which RpoS concentration is proportional to ppGpp inside cells, as would be expected from extrapolating data on one K-12 strain [9]. Not surprisingly, strains with undetectable RpoS have various ppGpp levels. Some strains, such as ECOR44,36,5,56,17,66 and 69 do exhibit a proportionality between the two measured entities, unlike ECOR14,55,58,65,54 and MG1655, which fall on a plateau with a limited amount of RpoS. This separation of responses in the ECORs was reinforced by multivariate analysis, which identified the relationship between three groups of strains (Figure 5b).

## Discussion

Sigma factors are high in the hierarchy of transcriptional regulators and are influenced by multiple environmental sensing pathways [45,46]. Molecules like ppGpp contribute to altering the pattern of transcription through sigma factors [15] and affect many important bacterial characteristics [20,47-49]. We address the question of the constancy of σ^S ^and ppGpp function across a species, beyond an individual lab strain.

The variation in σ^S ^levels and their physiological consequences across *E. coli *strains has been demonstrated earlier [28], and led to the idea of a trade-off between stress resistance (in high-RpoS strains) and nutritional capability (better in low-RpoS strains) [11]. This conclusion has been questioned [27]. Based on measurements of RpoS levels in six *E. coli *isolates these authors found a six-fold difference in RpoS level, with the highest RpoS only 1.49-times the MG1655 level. They noted that the trade-off hypothesis was originally based on only two high-RpoS strains in [28]. The variation of RpoS levels therefore needed a deeper analysis. Here we show that there is a much larger range of variation in σ^S ^amongst the ECOR isolates than Ihssen et al. found with fresh-water isolates. Further, we detected here sequence polymorphisms that would not have been observable in the earlier comparative genome hybridisation analysis [27]. Our conclusions are also consistent with results on RpoS variation in other laboratories [30,39] and recent indications that RpoS levels are highly variable within clinical populations of *E. coli *[50].

The variation in σ^S ^levels is not simply a result of differences in *rpoS *sequence. Variation in ppGpp was also evident in ECOR strains, revealing a possible diversifying influence on RpoS level and function [9,10]. ppGpp levels in ECOR strains showed dissimilarity particularly in response to carbon starvation. Variation in ppGpp levels was less with amino acid deprivation, consistent with greater variation in *spoT *than *relA *function. The conservation in *relA *function is not surprising, since the main role of RelA and the stringent response is to control the translational machinery of the cell in response to intracellular amino acid availability. This regulation is likely to be a universal need and hence widely conserved. In contrast, the response to extracellular nutrient availability and carbon starvation, mediated through *spoT*, is subject to fluctuating environmental inputs.

The *spoT *gene is central to the ppGpp-dependent response of bacteria to changes in growth rate and nutrient starvation [20] so it was interesting to find that it is subject to polymorphism both in laboratory strains [21] and ECOR isolates of *E. coli*. It is probably no accident that *spoT *variations were already noted in some lab lineages [51]. Further genomic comparisons in a BLAST search followed by a global alignment showed that 15 of 50 *E. coli *commensal and pathogenic strains currently in the sequence database have one or two amino acid substitutions in SpoT and two K-12 derivatives carry a QD insertion at position 84, the same insertion that is present in MC4100 [21]. In contrast, we found variation in only four out of 50 RelA sequences and three of them have only a single amino acid substitution between similar amino acids. Distinct mutations in *spoT *were also found in *E. coli *after thousands of generations of laboratory growth on glucose [52], suggesting *spoT *is subject to selection under repeat-batch culture conditions as well.

The strain variation in the concentration of ppGpp was more extensive than the genetic variation in *spoT*. Our results suggests that, as with *rpoS*, differences in ppGpp between natural isolates can be due to polymorphism in extragenic regulatory genes or in stress signal processing, as well as polymorphisms in *spoT *itself. For example, the steady-state level of ppGpp is increased in a *cgtA *mutant [53], but the accumulation of ppGpp during amino acid starvation is not affected, exactly as we find in some ECOR strains. CgtA interacts with SpoT and is thought to maintain low ppGpp levels when bacteria are growing in a nutrient-rich environment [54]. Further work on genomic and signal processing changes is needed to define all the influences leading to ppGpp variation in ECOR strains.

Traxler *et al*. have recently shown that increasing concentrations of ppGpp during the progression of amino acid limitation precisely activate genes related to the Lrp and RpoS regulons at a different stages [55]. According to these authors full induction of RpoS-dependent genes requires high concentrations of ppGpp. However, accumulation of RpoS is not due simply to increased ppGpp, once a ppGpp^0^strain still accumulates almost normal amounts of RpoS, although with a considerable delay [9,25]. It is therefore conceivable that as an alternative to ppGpp regulation another redundant mechanism operates to induce RpoS. This redundancy may explain the difficulty in establishing a clear relationship between ppGpp and RpoS and the consequent imperfect relation between ppGpp and RpoS described here. This is even more true for a heterogeneous set of strains as the ECOR collection, with its wide genetic heterogeneity.

Due to the number of strains tested, a growth-independent system for eliciting starvation was used to induce *relA *and *spoT*-dependent ppGpp accumulation. Hence the serine analogue SH and glucose analogue α-MG were used to induce amino acid and carbon limitation respectively. It is believed that α-MG acts through competition with glucose, thus limiting its utilisation. However, a recent study challenged this idea and proposed an alternative mechanism for α-MG toxicity resulting in growth arrest [56]. This explanation is based on the toxicity of α-MG phosphate, which accumulates in the cytoplasm. Nevertheless, whether growth arrest is caused by α-MG toxicity and/or competition with glucose, ppGpp accumulation due to α-MG is dependent on SpoT, because it occurs in both wild-type and *relA *mutants [44]. Furthermore, ppGpp accumulation following phosphate exhaustion with selected ECOR strains resulted in similar differences to the ones observed for α-MG treatment (results not shown).

As described for the *spoT*^+ ^and *spoT *variants of *E. coli *K12 [21], the nature of the *spoT *allele present in *E. coli *simultaneously influences the level of σ^S^, stress resistance and nutritional capabilities of *E. coli*. The environmental influence on ppGpp regulation is affected by the same dichotomy already observed and discussed for RpoS [11], namely the fluctuating needs of the cell in response to nutrient limitation and stress resistance. Indeed, the variation in *spoT *resembles the polymorphisms in *rpoS*, which are, if anything, even more extensive [26,39]. These new results suggest that one or more of the genes involved in ppGpp synthesis and degradation is subject to the same kind of selective pressures as is *rpoS*. In this respect, *spoT *and *rpoS *are both involved in SPANC balancing within a bacterium in response to changes in the immediate environment and hunger for nutrients.

## Conclusions

Two of the cellular components that control the allocation of transcriptional resources are strain-specific, since ppGpp and σ^S ^levels are potentially non-uniform in *E. coli *under identical growth conditions. A significant complication in the systems biology of *E. coli *is that even the regulatory relationship between ppGpp and RpoS is non-uniform across the species. The data from K-12 studies suggests ppGpp should stimulate RpoS synthesis, but the level of RpoS is not equally stimulated by high ppGpp in all ECOR isolates. As shown in Figure 5, there appear to be three groups of strains based on ppGpp/RpoS relationships, and in only one of these there is a discernible proportionality between ppGpp and RpoS concentrations. So not only is there likely to be variation in individual components, but also variation in the interaction of components of global networks. The new results suggest that the genes involved in ppGpp synthesis and degradation are also subject to the same kind of selective pressures as is *rpoS*. This has major consequences for the universality of the pattern of expression of hundreds of genes controlled directly or indirectly (by competition) at the level of RNA polymerase. The species-wide variation in the cellular concentration of two global directors of gene expression has significant implications for systems biology, because these regulators control many metabolic genes as well as gene expression networks [5,14]. Equally importantly, many of the numerous traits controlled by ppGpp [20,47-49] are also likely to be subject to strain variation.

## Methods

### Strains and media

The origins of the ECOR strains is described in [31] and the reference K-12 strain MG1655 was used for comparisons.

T-salts is a Tris-buffered minimal medium supplemented with different concentrations of glucose and KH_2_PO_4 _[18]. Minimal medium A (MMA) and L-agar plates were as in [57].

### Sequence analysis

The *rpoS *gene from different ECOR strains was amplified using the "universal" primer pair RpoS-F2 (5'-CCATAACGACACAATGCTGG) and RpoS-R2 (5'-CGACCATTCTCGGTTTTACC). PCR products were purified directly with Wizard DNA Preps DNA purification system (Promega). The nucleotide sequence of the *rpoS *gene was determined using either primer RpoS-F1 (5'- TGATTACCTGAGTGCCTACG) or RpoS-F2 for the first half and primer RpoS-I (5'- CTGTTAACGGCCGAAGAAGA) for the second half of gene.

For the sequencing of the *spoT *ORF, DNA was amplified by PCR using primers *spoT*F1 (5'-CAGTATCATGCCCAGTCATTTCTTC) and *spoT*R2 (5'-GGTAGTACTGGTTTCGCCGTGCTC). Sequencing analysis of both DNA strands were performed with primers *spoT*F1, *spoT*F2 (5'-AAAAGCGTCGCCGAGCTGGTAGAGG), *spoT*F3 (5'-TGATCGGCCCGCACGGTGTGCCGG), *spoT*F5 (5'-TGATCGGCCCGCACGGTGTGCCGG), *spoT*R1 (5'-TGCACCATCGCCATAATCATCTTGC), *spoT*R2 and *spoT*R3 (5'-CTTGATTTCGGTGATGAACTCCTG). All sequence reactions were done at the Australian Genome Research Facility.

### ppGpp assay

ppGpp was extracted from cells growing at 37°C in minimal medium containing 100 μCi/ml ^32^P-KH_2_PO_4_. For ppGpp extraction from C-starved ECOR strains, exponentially-growing cells were resuspended in T-salts supplemented with 0.1% glucose, 0.25 mM ^32^P-KH_2_PO_4 _and all 20 amino acids (30 μg/ml each) and grown for another 60 minutes. Methyl α-glucoside (α-MG) was then added at a final concentration of 2% and samples were withdrawn after 30 minutes in the single-point experiments or at several time intervals in the kinetic experiments.

Extraction of ppGpp from amino acid-starved cells was as above except that amino acid starvation was started by adding 1 mg/ml serine hydroxamate (SH) to the cultures.

The labelled samples were mixed immediately with 0.5 volume of cold formic acid and stored overnight at -20°C. The extracts were centrifuged for 5 minutes at 10,000 rpm to precipitate cell debris, and 3-5 μl were applied to PEI-cellulose TLC-plates. The labelled nucleotides were resolved by one-dimensional TLC using 1.5 M KH_2_PO_4 _as solvent. The amounts of ppGpp on the chromatograms were estimated by measuring the radioactivity of the spots in a Phosphor-Imager (Molecular Dynamics) and calculating the level of ppGpp relative to that of GTP + ppGpp [58]. The densitometric analysis was performed with the help of the Image J free software (available at http://rsb.info.nih.gov/ij/).

### Steady-state growth conditions in chemostats

T-salts supplemented with 0.02% glucose and 1.0 mM KH_2_PO_4 _was used to set up a 80 ml chemostat culture as described [32]. The dilution rate was set to 0.1 h^-1^. Daily samples were taken to monitor the *rpoS *status of members of the population. The *rpoS *status was determined by diluting the culture, growing the colonies on LB plates and staining with iodine (see below).

### Detection of *rpoS *status by iodine staining

The level of *rpoS *was qualitatively assessed by staining glycogen with an iodine solution as described [59]. Patches of bacteria or diluted chemostat samples were grown overnight on L-agar plates, stored at 4°C for 24 h and then flooded with iodine. The intensity of the brown colour varies according to the level of σ^S ^in the cell [28,60]. *rpoS*^+ ^strains stain brown to dark brown.

### Quantitation of RpoS blots

Bacteria cultures were grown overnight in LB medium at 37°C. LB medium possesses a limiting amount of amino acids that serve as main carbon sources. *E. coli *stops growing following overnight growth due to carbon depletion [61]. Culture volumes corresponding to 2. 10^9 ^cells were then centrifuged, resuspended in 200 μl application buffer (0,5 M Tris-HCl, 2% SDS, 5% 2-mercaptoethanol, 10% glycerol and 0,01% bromophenol blue) and boiled for 5 minutes. Proteins were resolved by SDS-PAGE in a 12,5% gel and transferred to a nitrocellulose membrane (GE HealthCare) by capillary force. Following blocking with 5% skim milk, the membrane was incubated with 2,000-fold diluted monoclonal anti-RpoS antibodies (Neoclone) and 20,000 fold diluted peroxidase conjugated anti-mouseIgG (Pierce). The Super Signal West Pico kit (Pierce) was used to detect the RpoS bands as recommended by the manufacturer. The membrane was exposed to X-ray films for various periods of time and the signal intensities on the autoradiograms were scanned and computed using the Image J software.

## Authors' contributions

TF conceived and designed the study, wrote and corrected the manuscript. HFG, TB and KP carried out the experimental work. BS performed experiments, conceived and designed the study, wrote and corrected the manuscript. All authors read and approved the final version of this manuscript.
